# Deeply jaundiced: Not so surgical after all

**DOI:** 10.4102/sajid.v38i1.559

**Published:** 2023-11-20

**Authors:** Wesley P. du Plessis, Sa’ad Lahri, Keethal Somers, Tamsin Lovelock

**Affiliations:** 1 Division of General Internal Medicine, Faculty of Medicine and Health Sciences, Tygerberg Hospital, Stellenbosch University, Cape Town, South Africa; 2 Division of Emergency Medicine, Faculty of Medicine and Health Sciences, Tygerberg Hospital, Stellenbosch University, Cape Town, South Africa; 3 Division of Infectious Diseases, Faculty of Medicine and Health Sciences, Tygerberg Hospital, Stellenbosch University, Cape Town, South Africa

**Keywords:** leptospirosis, severe leptospirosis, jaundice, travel, South Africa

## Abstract

Leptospirosis is an under-recognised disease in sub-Saharan Africa and the diagnosis requires a high index of suspicion. This case report highlights the protean manifestations of leptospirosis. Leptospirosis should be considered in any patient presenting with fever and jaundice, especially when there has been a history of occupational or recreational exposure to water, soil or rodents.

**Contribution:**

This case report describes a typical case of leptospirosis, which often presents as a diagnostic dilemma.

## Background

Leptospirosis is a common bacterial zoonotic disease worldwide with approximately 1 million cases yearly.^1^ There was an estimated annual incidence of 0.40 cases per 100 000 population in the Western Cape between 2010 and 2019.^2^ A recent outbreak in a South African prison revealed leptospira infection in 66.7% of rodents,^3^ suggesting that leptospirosis remains under-reported and under-recognised.^4^ The disease is frequently overlooked because of the high burden of other illnesses such as human immunodeficiency virus (HIV), tuberculosis and malaria in developing countries.^5^ Leptospirosis disproportionately affects vulnerable communities with poor living or working conditions and has been identified as a neglected tropical disease by the World Health Organization.^5^

Leptospirosis is caused by spirochaetes of the genus *Leptospira.*^6^ Humans become infected when they encounter the urine of infected animals either directly or via contaminated water or soil.^3^ Many animal species are reservoirs for pathogenic leptospires including rodents, domestic animals and livestock.^2,4^ Leptospirosis varies from mild, self-limiting or asymptomatic infection to life-threatening multisystem illness.^7^

## Case presentation

A previously well 36-year-old man presented to the medical emergency department at Tygerberg Hospital with a 3-day history of nausea, vomiting, headache, photophobia, fever, loss of appetite, abdominal pain and bloody diarrhoea. The patient, a Malawian national, works as a gardener and lives with his wife and children in a sandy informal settlement infested with rodents. He reported a significant ethanol history, which included binge drinking on weekends. As a result of an initial language barrier, the travel history was unclear, but any recent travel history was subsequently disproven. On initial assessment, the patient was tachycardic, hypotensive and pyrexial with a heart rate of 128 beats per minute (bpm), blood pressure of 69/35 mmHg and temperature of 38.1 °C. He was profoundly jaundiced with prominent conjunctival suffusion. The respiratory examination was notable for left lower zone crackles and generalised abdominal tenderness and guarding on abdominal examination.

The patient was started on vasopressors after an inadequate response to fluid resuscitation and empiric antibiotic therapy with ceftriaxone was initiated. Blood and urine cultures and a lumbar puncture were performed on the day of admission. The chest radiograph revealed no abnormalities. Blood results were remarkable for elevated C-reactive protein of 349 mg/L (0 mg/L – 10 mg/L), a leukocytosis of 20.21 × 10^9^/L (3.92 – 10.4 × 10^9^/L), renal impairment with blood urea nitrogen of 20.8 mmol/L (2.1 mmol/L – 7.1 mmol/L) and creatinine of 331 μmol/L (64 μmol/L – 104 μmol/L), conjugated hyperbilirubinemia of 109 μmol/L (0 μmol/L – 3 μmol/L) and total bilirubin of 126 μmol/L (5 μmol/L – 21μmol/L), an ALT of 35 U/L (10 U/L – 40 U/L) and aspartate aminotransferase (AST) of 98 U/L (15 U/L – 40 U/L), sodium of 135 mmol/L (136 mmol/L – 145 mmol/L), albumin of 28 g/L (35 g/L – 52 g/L) and thrombocytopenia of 38 × 10^9^/L (171 – 388 × 10^9^/L). A disseminated intravascular coagulation (DIC) screen revealed an international normalised ratio (INR) of 1.23, fibrinogen of 9.0 g/L (2 g/L – 4 g/L) and D-dimer of 1.05 mg/L (0.00 mg/L – 0.25 mg/L). Cerebrospinal fluid revealed a protein of 0.41 g/L (0.15 g/L – 0.45 g/L), glucose of 3.7 mmol/L, cell count showed 2 lymphocytes, 40 erythrocytes, no polymorphs and a negative gram stain and culture. The patients HIV serology was negative. The computed tomography imaging of the abdomen revealed peri-appendiceal fluid, but the surgical review was inconsistent with appendicitis.

After referral to the Internal Medicine department, further investigations included a malaria rapid diagnostic test, thin and thick blood smears and serology for leptospirosis. The patient demonstrated a significant clinical improvement within 24 h of ceftriaxone initiation and was weaned from vasopressor therapy. Blood and urine cultures revealed no growth, malaria rapid antigen and urine *Legionella* antigen testing were negative. Serology for hepatitis A, B and C was also negative. On day eight, the *Leptospira* IgM was positive. The patient was discharged after 10 days of ceftriaxone when haemodynamically stable, apyrexial and mobilising in the ward. His renal function had normalised, and serum bilirubin was on a downward trend. An infectious diseases outpatient appointment was planned but not kept. A telephonic follow-up was carried out approximately 2 months post-discharge and the patient reported that he felt entirely well.

## Differential diagnosis

Leptospirosis has a varied clinical presentation and may mimic many other illnesses. The presence of fever and a possible history of travel requires the consideration of infectious causes such as malaria or enteric fever. The prominent jaundice and abdominal pain plus a history of ethanol use made it essential to also consider non-infectious entities such as acute alcoholic hepatitis or pancreatitis. A more comprehensive list of differential diagnoses is detailed in Table 1.

**TABLE 1:** Differential diagnosis for the patient with fever, jaundice, abdominal pain and multi-organ dysfunction.

Infectious	Non-infectious
Malaria	Any cause of biliary obstruction: tumour, lymphadenopathy
Enteric fever	Cholecystitis
Pyogenic and/or amoebic liver abscess	Appendicitis
Sepsis with multi-organ failure	Drug and/or toxin-induced liver injury
Tick bite fever	Thrombotic microangiopathies
Viral haemorrhagic fevers	Porphyria

## Discussion

The patient presented with a clinical syndrome of fever, jaundice and circulatory collapse.

Prominent gastrointestinal symptoms including abdominal pain, vomiting and diarrhoea resulted in an initial assessment of appendicitis. Gastrointestinal symptoms are commonly found in leptospirosis and pain may be attributed to cholecystitis or pancreatitis, which is self-limiting.^8^ An abnormal lipase or amylase may be observed.^8,9^ In this case, abdominal examination findings were not in keeping with appendicitis or other surgical causes of fever and jaundice, prompting urgent consideration of a broader differential diagnosis.

Initial special investigations revealed multisystem involvement, including renal failure and thrombocytopenia. The possible travel history was an important consideration, and malaria was excluded. Other important considerations in the returning traveller would be diseases of public health importance, such as enteric fever and viral haemorrhagic fever syndromes. Tick bite fever should always be considered and empirically treated in patients with fever and multisystem disease when an alternative aetiology remains unclear. In retrospect, the patient’s living environment and occupation as risk factors for leptospirosis should have considered.

The symptoms of headache and photophobia suggest meningitis. The diagnosis of leptospirosis may be challenging in meningitis without jaundice and renal failure.^10^ The cerebrospinal fluid analysis was unremarkable; however, it is important to observe that aseptic meningitis is a common neurological manifestation of leptospirosis.^10,11^

Leptospirosis presents with a conjugated hyperbilirubinaemia out of proportion to derangements in the liver enzyme profile as noticed in our patient.^12^ In contrast, transaminitis is usually more prominent in the viral hepatitides. Imaging to exclude hepatic duct obstruction is appropriate in any patient with jaundice.

Leptospires have an affinity for the kidney and commonly cause renal injury.^8^ Acute kidney injury in leptospirosis can be multifactorial, including a prerenal component because of reduced intake and vomiting and acute tubular necrosis because of hypotension and sepsis. Renal failure in leptospirosis is a frequent cause of death in patients who develop oliguria without dialysis.^8^

Thrombocytopenia is present in 50% – 93% of leptospirosis cases and often predicts renal failure.^13^ The triad of thrombocytopenia, fever and renal failure suggests leptospirosis, malaria or dengue fever,^14^ but non-infectious aetiologies such as microangiopathic haemolytic anaemias are also possible. In our case, no fragments were observed on the peripheral blood smear and thrombocytopenia improved with antibiotic therapy.

Systemic inflammatory response syndrome together with infection is classified as sepsis.^15^ Leptospirosis causes endothelial damage in multiple organs and can cause a systemic inflammatory response syndrome, but unlike sepsis, the organ involvement is rapidly reversible.^16^ In this case, the patient had features suggestive of septic shock, with broad-spectrum antibiotics promptly initiated, the source of sepsis was sought.

Leptospirosis typically has two forms, a milder anicteric form that may resolve completely or progress to the more severe icteric form, which is characterised by multi-organ dysfunction.^17^ The severe form, Weil’s disease, is caused by *Leptospira interrogans* serovar icterohaemorrhagiae and is mainly found in rats.^18^ Human infection with leptospirosis is variable, and patients can be asymptomatic, have non-specific symptoms or develop complex febrile disease.^7^ The presence of conjunctival suffusion (conjunctival erythema without purulent exudate) on examination is an almost pathognomonic finding of leptospirosis, as it is not present with other infectious diseases but may not occur in all cases.^8^

The diagnosis of leptospirosis in this case was based on a positive *Leptospira* immunoglobulin M enzyme-linked immunoassay (IgM ELISA). Leptospirosis can be detected via several methods including microscopy, culture, serology and polymerase chain reaction (PCR). Microscopic agglutination testing is the gold standard but is resource intensive and not widely available. Culture requires extended incubation, which delays diagnosis, and although PCR has increased sensitivity, its lack of availability limits its usefulness. The IgM ELISA is more widely available, therefore much more commonly used and can detect antibodies by the fifth day of symptom onset.^19^

Management of leptospirosis includes antibiotic treatment and supportive care. Leptospira are susceptible to penicillin, tetracycline and ceftriaxone.^20^ Doxycycline can be used for mild disease and intravenous penicillin G or ceftriaxone for severe disease with multiorgan involvement.^21^

## Conclusion

This case report highlights the importance of considering leptospirosis in patients presenting with fever, jaundice and a possible history of exposure to rodents and contaminated water or soil, in impoverished settings with poorer living conditions. Leptospirosis has a varied clinical presentation and can mimic many other illnesses, including surgical diseases such as cholecystitis, appendicitis and pancreatitis. The protean manifestations of leptospirosis require a high index of suspicion for a timely diagnosis in order to institute appropriate management. Early recognition and treatment can prevent severe complications and improve patient outcomes. It is essential to raise awareness of this under-recognised disease to improve surveillance and public health interventions to mitigate the impact of leptospirosis in vulnerable populations.

## Teaching points

- Clinicians should consider leptospirosis in any patient presenting with fever and jaundice. The diagnosis should be considered early to prevent morbidity and mortality.
- The abdominal pain in leptospirosis may be severe and is often initially considered surgical in nature.
- Conjugated hyperbilirubinaemia without biliary obstruction and out of proportion to the transaminitis is a typical clinical feature of leptospirosis and conjunctival suffusion is pathognomonic but not always present.
- Living circumstances and occupation must be considered.

## References

[1] Costa F, Hagan JE, Calcagno J, et al. Global morbidity and mortality of leptospirosis: A systematic review. PLoS Negl Trop Dis. 2015;9(9):e0003898. 10.1371/journal.pntd.0003898 26379143 PMC4574773

[2] Gizamba JM, Paul L, Dlamini SK, Odayar J. Incidence and distribution of human leptospirosis in the Western Cape Province, South Africa (2010–2019): A retrospective study. Pan Afr Med J. 2023;44:121. 10.11604/pamj.2023.44.121.34244 37275293 PMC10237220

[3] Naidoo K, Moseley M, McCarthy K, et al. Fatal rodentborne leptospirosis in prison inmates, South Africa, 2015. Emerg Infect Dis. 2020;26(5):1033–1035. 10.3201/eid2605.191132 32310070 PMC7181914

[4] Allan KJ, Biggs HM, Halliday JE, et al. Epidemiology of leptospirosis in Africa: A systematic review of a neglected zoonosis and a paradigm for ‘one health’ in Africa. PLoS Negl Trop Dis. 2015;9(9):e0003899. 10.1371/journal.pntd.0003899 26368568 PMC4569256

[5] World Health Organization. Report of the second meeting of the Leptospirosis Burden Epidemiology Reference Group [homepage on the Internet]. Geneva: WHO; 2011 [cited 2023 Apr 10]. Available from: https://apps.who.int/iris/handle/10665/44588

[6] Adler B, De La Peña Moctezuma A. Leptospira and leptospirosis. Vet Microbiol. 2010;140(3–4):287–296. 10.1016/j.vetmic.2009.03.012 19345023

[7] De Vries SG, Visser BJ, Nagel IM, Goris MGA, Hartskeerl RA, Grobusch MP. Leptospirosis in sub-Saharan Africa: A systematic review. Int J Infect Dis. 2014;28:47–64. 10.1016/j.ijid.2014.06.013 25197035

[8] Haake DA, Levett PN. Leptospirosis in humans. In: Adler B, editor. Leptospira and leptospirosis. Current topics in microbiology and immunology. Berlin: Springer, 2015; p. 65–97. 10.1007/978-3-662-45059-8_5 PMC4442676 25388133

[9] O’Brien MM, Vincent JM, Person DA, Cook BA. Leptospirosis and pancreatitis: A report of ten cases. Pediatr Infect Dis J. 1998;17(5):436–438. 10.1097/00006454-199805000-00023 9613666

[10] Bandara AG, Kalaivarny G, Perera N, Indrakumar J. Aseptic meningitis as the initial presentation of *Leptospira borgpetersenii* serovar tarassovi: Two case reports and a literature review. BMC Infect Dis. 2021;21(1):488. 10.1186/s12879-021-06200-w 34044779 PMC8161910

[11] Panicker JN, Mammachan R, Jayakumar RV. Primary neuroleptospirosis. Postgrad Med J. 2001;77(911):589–590. 10.1136/pmj.77.911.589 11524519 PMC1757912

[12] Jiang K, Bazarbashi AN, Dahdal S, Voiculescu A, Khalaf N. The spiraling case of a yellow chef: Isolated hyperbilirubinemia. Case Rep Hepatol. 2018;2018:5876718. 10.1155/2018/5876718 PMC6126086 30210882

[13] Sharma J, Suryavanshi M. Thrombocytopenia in leptospirosis and role of platelet transfusion. Asian J Transf Sci. 2007;1(2):52. 10.4103/0973-6247.33447 PMC3168120 21938233

[14] Prabhu MV, Arun S, Ramesh V. Fever, thrombocytopenia, and aki-a profile of malaria, dengue, and leptospirosis with renal failure in a south Indian tertiary-care hospital. Clin Nephrol. 2016;86(S1):128–130. 10.5414/CNP86S118 27509584

[15] Dellinger RP, Levy MM, Rhodes A, et al. Surviving sepsis campaign: International guidelines for management of severe sepsis and septic shock, 2012. Intens Care Med. 2013;39(2):165–228. 10.1007/s00134-012-2769-8 PMC7095153 23361625

[16] Yilmaz H, Turhan V, Yasar KK, Hatipoglu M, Sunbul M, Leblebicioglu H. Characteristics of leptospirosis with systemic inflammatory response syndrome: A multicenter study. Ann Clin Microbiol Antimicrob. 2015;14(1):54. 10.1186/s12941-015-0117-x 26690093 PMC4687284

[17] Saif A, Frean J, Rossouw J, Trataris AN. Leptospirosis in South Africa. Onderstepoort J Vet Res. 2012;79(2):478. 10.4102/ojvr.v79i2.478 23327372

[18] Roczek A, Forster C, Raschel H, et al. Severe course of rat bite-associated Weil’s disease in a patient diagnosed with a new leptospira-specific real-time quantitative Lux-PCR. J Med Microbiol. 2008;57(5):658–663. 10.1099/jmm.0.47677-0 18436602

[19] Pinto GV, Senthilkumar K, Rai P, Kabekkodu SP, Karunasagar I, Kumar BK. Current methods for the diagnosis of leptospirosis: Issues and challenges. J Microbiol Methods. 2022;195:106438. 10.1016/j.mimet.2022.106438 35248601

[20] Goarant C. Leptospirosis: Risk factors and management challenges in developing countries. Res Rep Trop Med. 2016;7:49–62. 10.2147/rrtm.s102543 30050339 PMC6028063

[21] Rajapakse S. Leptospirosis: Clinical aspects. Clin Med. 2022;22(1):14–17. 10.7861/clinmed.2021-0784 PMC8813018 35078790

